# Taxogenomic status of phylogenetically distant *Frankia* clusters warrants their elevation to the rank of genus: A description of *Protofrankia* gen. nov., *Parafrankia* gen. nov., and *Pseudofrankia* gen. nov. as three novel genera within the family *Frankiaceae*

**DOI:** 10.3389/fmicb.2022.1041425

**Published:** 2022-11-08

**Authors:** Maher Gtari

**Affiliations:** USCR Bactériologie Moléculaire & Génomique, Institut National des Sciences Appliquées et de Technologie, Université de Carthage, Tunis, Tunisia

**Keywords:** *Frankiaceae*, bacterial genus boundaries, phylogenomics, pangenome, AAI, POCP, ANI, 16S rRNA gene

## Abstract

The genus *Frankia* is at present the sole genus in the family *Frankiaceae* and encompasses filamentous, sporangia-forming actinomycetes principally isolated from root nodules of taxonomically disparate dicotyledonous hosts named actinorhizal plants. Multiple independent phylogenetic analyses agree with the division of the genus *Frankia* into four well-supported clusters. Within these clusters, *Frankia* strains are well defined based on host infectivity range, mode of infection, morphology, and their behaviour in culture. In this study, phylogenomics, overall genome related indices (OGRI), together with available data sets for phenotypic and host-plant ranges available for the type strains of *Frankia* species, were considered. The robustness and the deep radiation observed in *Frankia* at the subgeneric level, fulfilling the primary principle of phylogenetic systematics, were strengthened by establishing genome criteria for new genus demarcation boundaries. Therefore, the taxonomic elevation of the *Frankia* clusters to the rank of the genus is proposed. The genus *Frankia* should be revised to encompass cluster 1 species only and three novel genera, *Protofrankia* gen. nov., *Parafrankia* gen. nov., and *Pseudofrankia* gen. nov., are proposed to accommodate clusters 2, 3, and 4 species, respectively. New combinations for validly named species are also provided.

## Introduction

The genus *Frankia* Brunchorst, 1886 (Approved Lists 1980) (Brunchorst, 1886; Becking, 1970; Skerman et al., 1980; Lechevalier and Lechevalier, 1989) is currently the solitary genus within the family *Frankiaceae* (Becking, 1970; Hahn et al., 1989; Normand et al., 1996; Stackebrandt et al., 1997) of the order *Frankiales* (Sen et al., 2014; Normand and Benson, 2015). The genus encompasses soil-inhabiting mesophilic actinomycetes mostly are able to fix dinitrogen and to establish symbiosis with pioneer and economically important plants, collectively named actinorhizal plants (Normand and Fernandez, 2019). Both in culture and the host root nodules, *Frankia* strains produce branched septate hyphae which, for most analysed strains, can carry multilocular sporangia, while the diazotrophic strains produce vesicles, where nitrogen fixation occurs (Normand et al., 2014; Gtari et al., 2019). Based on the 16S rRNA gene phylogeny (Normand et al., 1996), which was further substantiated by internal transcribed spacer (ITS) 16S-23S rRNA genes (Ghodhbane-Gtari et al., 2010), multilocus sequence analysis (MLSA) (Nouioui et al., 2011; Persson et al., 2011; Sen et al., 2014; Gtari et al., 2015; Pozzi et al., 2018), amplified fragment length polymorphism (AFLP) (Bautista et al., 2011) and whole-genome analyses (Gtari et al., 2019; Nouioui et al., 2019b), four phylogenetic clusters are consistently delineated within the genus, grouping strains with similar cultural behaviour, morphology, host range, and mode of infection (Benson and Silvester, 1993). Cluster 1 members colonise species of *Alnus* (*Betulaceae*), *Allocasuarina* and *Casuarina* (*Casuarinaceae*), and *Comptonia* and *Myrica* (*Myricaceae*), while cluster 2 contains strains that infect *Coriariaceae*, *Datiscaceae*, *Dryadoideae* (*Rosaceae*), and *Ceanothus* (*Rhamnaceae*). Cluster 3 contains *Frankia* associated with *Elaeagnaceae*, *Colletieae* (*Rhamnaceae*), *Morella* (*Myricaceae*), and *Gynmnostoma* (*Casuarinaceae*). Members of cluster 3 have been also occasionally isolated from *Alnus*, *Allocasuarina*, *Casuarina*, *Ceanothus*, and *Dryadoideae* root nodules (Benson and Dawson, 2007). Finally, cluster 4 includes asymbiotic *Frankia* strains, which are unable to fix nitrogen and/or to re-infect their host plants.

Prior to the genomic era, bacterial taxonomy relied on the polyphasic approach (Colwell, 1970) which integrates morphological, metabolic, and chemotaxonomic makers, 16S rRNA phylogeny, and pairwise dissimilarity. For ambiguous situations, the gold standard DNA–DNA hybridization (DDH) was used for drawing conclusions on species delineation (Wayne et al., 1987). For the genus *Frankia*, problems of applying traditional bacteriological techniques persisted due primarily to the high proportion of uncultivable strains and, secondly, to the very slow growing rate of most cultivated strains. Metabolic behaviours and wet-lab experimental DNA relatedness were, thus, inconsistent when the polyphasic taxonomic approach was applied in the case of the genus *Frankia* (Normand and Fernandez, 2008; Gtari et al., 2013).

Taking advantage of incorporating Taxogenomic and omniLog^®^ phenoarray into the polyphasic approach (Gtari et al., 2019), 13 species with validly published names have been described based on accepted thresholds for bacterial species delineation, i.e., 99.0% (with a maximum probability of error of 1.0%) for 16S rRNA similarities (Meier-Kolthoff et al., 2013), 70% for digital DDH (Auch et al., 2010), and 95% for average nucleotide identity (ANI) (Konstantinidis et al., 2006). Ten of these species are facultative symbiotic species from clusters 1, 2, and 3 and include the type species *Frankia alni* (Nouioui et al., 2016), *Frankia canadensis* (Normand et al., 2018), *Frankia casuarinae* (Nouioui et al., 2016), and *Frankia torreyi* (Nouioui et al., 2019a) of cluster 1; *Frankia coriariae* (Nouioui et al., 2017b) of cluster 2; and *Frankia elaeagni* (Nouioui et al., 2016), *Frankia discariae* (Nouioui et al., 2017d),*Frankia irregularis* (Nouioui et al., 2018b), *Frankia soli* (Gtari et al., 2020), and *Frankia colletiae* (Nouioui et al., 2022) of cluster 3. Cluster 4 includes *Frankia asymbiotica* (Nouioui et al., 2017c), *Frankia inefficax* (Nouioui et al., 2017a), and *Frankia saprophytica* (Nouioui et al., 2018a). Additionally, four candidate species are also defined to accommodate uncultured taxa: *Candidatus* Frankia datiscae (Persson et al., 2011) and *Candidatus* Frankia californiensis (Normand et al., 2017) from cluster 2, as well as *Candidatus* Frankia alpina (Pozzi et al., 2020) and *Candidatus* Frankia nodulisporulans (Herrera-Belaroussi et al., 2020) from cluster 1.

Bacterial classification at higher taxonomic ranks relies primarily on phylogenetic systematics (Ludwig and Klenk, 2005; Oren and Garrity, 2014) which has been greatly improved through phylogenomics (Dagan, 2011; Oren and Garrity, 2014; Hugenholtz et al., 2021). Genomic criteria for demarcating genus boundaries include conserved indels and proteins signatures (Naushad et al., 2014), comparative pangenome (Caputo et al., 2019), average amino acid identity (AAI) (Konstantinidis and Tiedje, 2007), percentage of conserved proteins (POCP) (Qin et al., 2014), and ANI (Barco et al., 2020). The gold standard overall genome related indices (OGRI) for genus demarcation remains pending inquiry (Sant’Anna et al., 2019).

The commonplace availability of genomic tools and algorithms for phylogenetic purposes and OGRI has motivated our interest to review the taxonomic structure of the genus *Frankia* in relation to the evolutionary history of its well-known phylogenetic clusters. The results obtained in the present study support the taxonomic elevation of each of the four clusters to the rank of the genus. Hence, three novel genera, *Protofrankia* gen. nov., *Parafrankia* gen. nov., and *Pseudofrankia* gen. nov., to accommodate clusters 2, 3, and 4, respectively, with 9 related new combinations, are proposed.

## Materials and methods

Complete and draft genomes for type strains and candidate species together with other selected published genomes covering current *Frankia* diversity were used in this study and are listed in Table 1.

**TABLE 1:** Genome characteristics for strains used in the present study.

	Strains	Scaffold	Genome size bp	Gene	G + C%	Protein coding gene	RNA	rRNA	tRNA	CRISPR	COG	Enzyme	KEGG	HTG
Cluster 1	*Frankia alni* ACN14a^T^	1	7,497,934	6,795	72.83	6,723	72	6	46	11	3,434	1,465	1,437	429
	*Frankia torreyi* CpI1^T^	153	7,624,758	6,448	72.43	6,373	75	5	47	4	3,634	1,362	1,347	41
	*Frankia canadensis* ARgP5^T^	568	7,673,585	6,894	72.39	6,799	57	3	52	7	4,642	1,393	1,343	0
	*Candidatus* Frankia alpina	669	5,504,816	5,659	71.57	5,574	57	8	47	2	3,435	1,029	1,011	234
	*Candidatus* Frankia nodulisporulans	612	4,882,652	4,602	71.61	4,528	55	6	46	1	2,973	988	972	223
	*Frankia* sp. QA3^T^	1	7,590,853	6,546	72.59	6,493	53	4	46	8	3,669	1,389	1,403	132
	*Frankia* sp. ACN1ag	90	7,521,104	6,312	72.5	6,247	65	5	45	3	3,626	1,353	1,337	6,055
	*Frankia* sp. AvcI.1	77	7,741,902	6,530	72.74	6,470	60	5	46	5	3,672	1,412	1,396	45
	*Frankia casuarinae* CcI3^T^	1	5,433,628	4,621	70.08	4,548	73	6	46	7	2,438	1,211	1,160	151
	*Frankia* sp. CeD	120	5,004,595	4,466	70.2	4,403	63	7	45	1	2,372	1,092	1,062	3
	*Frankia* sp. KB5	420	5,455,564	4,675	70.11	4,622	53	6	45	2	2,416	1,107	1,093	243
Cluster 2	*Frankia coriariae* BMG5.1^T^	102	5,789,716	5,333	70.24	5,277	56	3	45	7	2,491	1,044	1,073	593
	*Frankia* sp. BMG5.30	94	5,818,019	5,034	70.21	4,976	58	5	45	5	2,662	1,132	1,153	15
	*Candidatus* Frankia datiscae Dg1	1	5,323,186	4,254	70.04	4,202	52	6	44	4	2,452	1,090	1,078	1
	*Candidatus* Frankia californiensis Dg2	2,738	5,896,456	7,108	68	7,022	65	4	39	8	4,102	988	978	893
Cluster 3	*Frankia elaeagni* BMG5.12^T^	135	7,589,313	6,342	71.67	6,253	89	5	51	1	3,516	1,390	1,356	5,654
	*Frankia discariae* BCU110501^T^	194	7,891,711	6,839	72.39	6,742	97	8	47	7	3,671	1,399	1,350	954
	*Frankia irregularis* G2^T^	83	9,537,992	7,873	70.95	7,789	84	9	47	3	4,538	1,635	1,605	7,525
	*Frankia soli* Cj^T^	289	8,032,739	6,296	71.73	6,244	52	5	45	3	3,609	1,390	1,378	531
	*Frankia colletiae* Cc1.17^T^	195	8,361,025	6,392	71.44	6,343	49	0	47	0	3,870	1,440	1,424	335
	*Frankia* sp. Ea1.12	749	8,022,419	7,429	71.67	7,308	63	4	57	1	4,995	1,412	1,371	200
	*Frankia* sp. BMG5.11	219	11,255,272	10,281	69.87	10,106	122	7	109	4	7,207	2,369	2,326	2,876
	*Frankia* sp. EUN1f	2	9,322,173	7,833	70.82	7,775	58	9	47	12	4,380	1,659	1,632	7
	*Frankia* sp. R43	31	10,442,526	8,596	70.91	8,523	73	9	45	4	4,797	1,775	1,721	1,400
	*Frankia* sp. EI5c	155	6,617,243	5,515	72.19	5,452	63	4	46	2	3,260	1,318	1,302	215
Cluster 4	*Frankia inefficax* EuI1c^T^	1	8,815,781	7,262	72.31	7,205	57	9	46	1	4,499	1,764	1,738	3
	*Frankia asymbiotica* M16386^T^	174	9,435,764	7,904	71.97	7,821	83	3	70	5	4,483	1,586	1,562	651
	*Frankia saprophytica* CN3^T^	2	9,978,592	8,411	71.81	8,332	79	5	68	6	4,930	1,662	1,633	211
	*Frankia* sp. EUN1h	129	9,910,952	7,477	71.86	7,405	72	2	68	4	4,617	1,586	1,562	14
	Frankia sp. BMG5.36	280	11,203,906	8,330	71.26	8,250	80	11	67	3	4,966	1,722	1,701	1,287
	*Frankia* sp. DC12	1	6,884,336	5,933	71.93	5,858	75	9	46	2	3,162	1,313	1,254	89

### Phylogenetic analysis

Phylogenetic analyses based on 16S rRNA gene sequences were carried out using the GGDC web server adapted to single genes (Meier-Kolthoff et al., 2013). Maximum-likelihood (ML) and maximum-parsimony (MP) trees were inferred with RAxML (Stamatakis, 2014) and tree analysis new technology (TNT) (Goloboff et al., 2008), respectively. For ML, rapid bootstrapping was used in conjunction with the autoMRE bootstrapping criterion (Pattengale et al., 2010), followed by a search for the best tree. For MP, 1,000 bootstrapping replicates were used in conjunction with tree-bisection-and-reconnection branch swapping and 10 random addition-sequence replicates. The sequences were checked for compositional bias using the *χ*^2^ test as implemented in phylogenetic analysis using parsimony (PAUP)* (Swofford, 2002).

The whole-genome-based taxonomic analysis was performed through the Type Strain Genome Server^1^ (Meier-Kolthoff and Göker, 2019; Meier-Kolthoff et al., 2022). Pairwise genomic comparisons were calculated and intergenomic distances were inferred under the algorithm “trimming” and distance formula d5 using 100 distance replicates in FastME 2.0 (Lefort et al., 2015).

The pangenome analysis was performed using Roary (Page et al., 2015) implemented in the “Pan” module of the Prokaryotic Genomics and Comparative Genomics Analysis Pipeline (PGCGAP v1.0.21) (Liu et al., 2020). Single-copy analysis of core proteins, alignment of sequences, sequence concatenation, best model, and phylogenetic tree reconstruction based on 303 single copies of core proteins were performed with the “CoreTree” module of PGCGAP v1.0.21.

### Overall genome related indices calculations

Overall genome related indices, including pairwise ANI, were calculated through the IMG/M data management and analysis system (Varghese et al., 2015). Pairwise AAI was calculated with the EzAAI tool (v1.1) (Kim et al., 2021) with default settings, which use MMSeqs2 for protein comparisons and consider a minimum query coverage of 50% and a minimum identity of 40% for AAI calculations. Pairwise POCP was calculated according to Qin et al.’s (2014) findings, following the steps described by Adamek et al. (2018) and Margos et al. (2018). Reciprocal BlastP (Altschul, 1997) for each pair of genomes used an E-value of < 1 × 10^–5^, > 40% sequence identity and > 50% of the query sequence. The pairwise POCP value was then deduced as [(C1 + C2)/(T1 + T2)] × 100, where C1 and C2 represent the conserved proteins numbers between the genome pair, while T1 and T2 are the total numbers of compared proteins in each genome (Qin et al., 2014).

## Results and discussion

### Phylogenetics and phylogenomics

**FIGURE 1:**
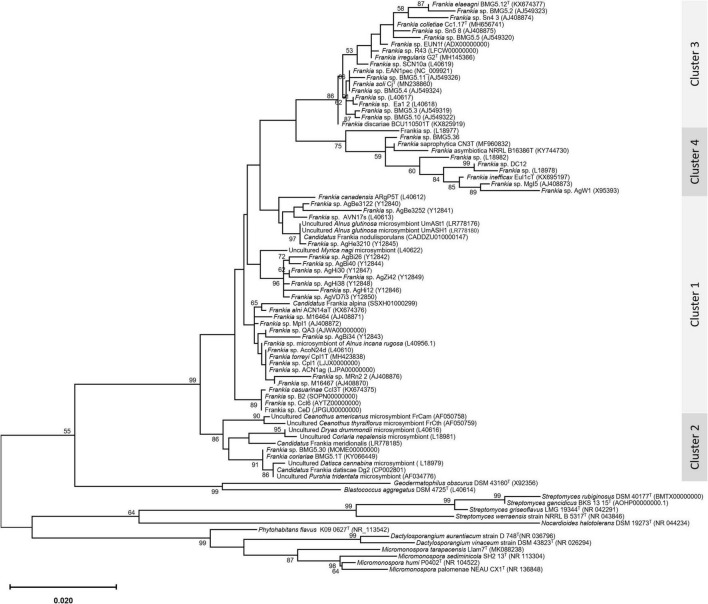
A maximum-likelihood tree based on the 16s rRNA gene sequences of 72 members of the genus *Frankia*. The maximum-likelihood (ML) tree was inferred using the GTR + GAMMA model and rooted by midpoint rooting, and the branches are scaled in terms of the expected number of substitutions per site. The numbers above the branches support values when larger than 60% bootstrapping.

**FIGURE 2:**
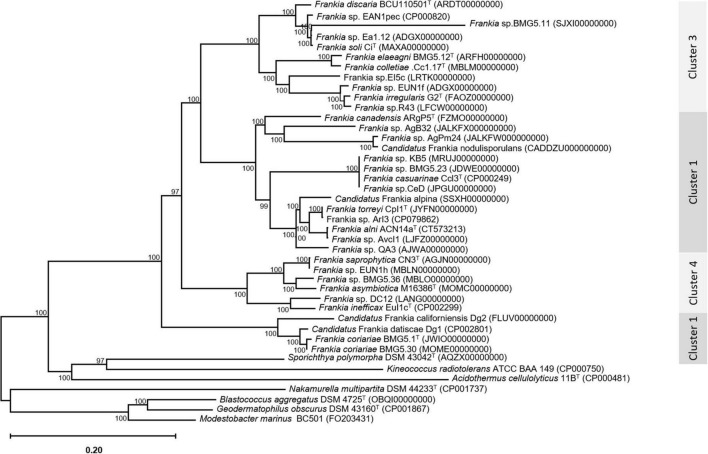
A maximum-likelihood tree inferred by the module “CoreTree” of PGCGAP v1.0.21 (Liu et al., 2020) with the best-fit model JTT + F + R4 and based on the concatenation of single-copy conserved proteins. The midpoint-rooted phylogenetic tree of single-copy core proteins.

**FIGURE 3:**
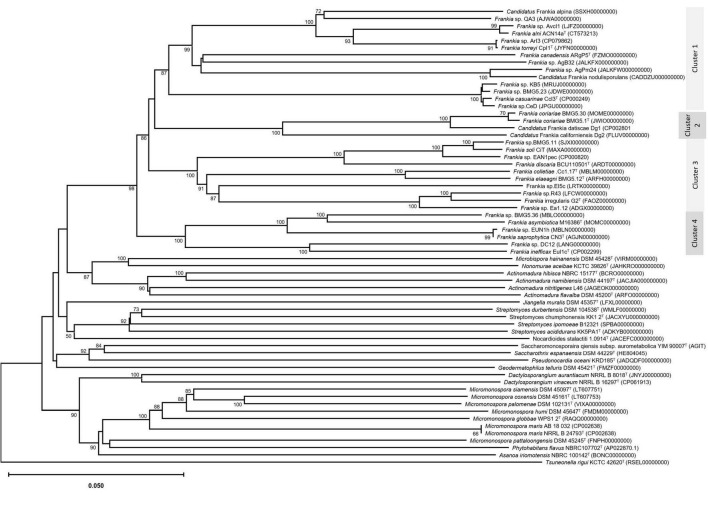
Phylogenomic tree based on genome sequences in the Type Strain Genome Server (TYGS) tree inferred in FastME 2.1.6.1 (Meier-Kolthoff and Göker, 2019) based on genome BLAST distance phylogeny (GBDP). The branch lengths are scaled based GBDP distance formula d5. The tree was rooted at the midpoint (Meier-Kolthoff et al., 2013). GBDP pseudo-bootstrap support values > 60% from 100 replications, with average branch support of 93.7% were shown.

An update of the 16S rRNA phylogeny was, here made available using the largest possible dataset of sequences (n = 72) from all type strains and candidate species, and those available in public databases or extractable from genome sequences providing a size of ≥ 900 nt (Figure 1). A more robust phylogenetic history was obtained using a single copy of core proteins (Figure 2) and whole-genome sequences (Figure 3), as shown by higher bootstrap and posterior probability values supporting the branching for the four clusters. Otherwise, in congruence with Salam et al. (2020), the most closely related *Actinomycetia* to *Frankiaceae* are *Acidothermus* (*Acidothermaceae* and *Acidothermales*) and *Jatrophihabitans* (*Jatrophihabitantaceae* and *Jatrophihabitantales*).

The trees obtained, in this study, showed broadly similar patterns and topology with those previously reported for the 16S rRNA gene (Normand et al., 1996), ITS 16S-23S rRNA (Ghodhbane-Gtari et al., 2010), AFLP (Bautista et al., 2011), or combined data sets; 16S rRNA and *gln*A (Clawson et al., 2004), *Gyr*B, *gln*II, and *nif*H (Nouioui et al., 2011), *atp*D, *dna*A, *fts*Z, *pg*k, and *rpo*B (Sen et al., 2014; Gtari et al., 2015; Nouioui et al., 2017a,b, 2018a,2018b,2019a; Pozzi et al., 2018), as well as 50 (Sen et al., 2014; Persson et al., 2015) or up to 200 gene sequences (Gtari et al., 2019; Nouioui et al., 2019b). Alongside the trees’ robustness and topology congruencies for phylogenetic splitting, no ambiguous or shifting affiliations between clusters were observed for any of the studied strains regardless of the algorithms or the extent of the genomic region used for inferring *Frankia* evolutionary history.

The relative positioning of each of the four *Frankia* clusters, and the timeline assumed for their separation, have been for a long a source of debate. While 16S rRNA (Normand et al., 1996), ITS 16S-23S rRNA (Ghodhbane-Gtari et al., 2010), AFLP (Bautista et al., 2011), and MLSA (Nouioui et al., 2011) based phylogenies placed cluster 4 at the base of the tree, followed by cluster 3 and then clusters 1 and 2 which form sister groups, and other MLSA studies (Pozzi et al., 2018) or concatenation of proteins (Sen et al., 2014; Gtari et al., 2015; Persson et al., 2015) showed cluster 2 as basal, followed by cluster 4 and then the symbiotic cluster 3, and finally, cluster 1 as the most derived. Both situations were here obtained based on the whole genome (Figure 3) and on core proteins (Figure 2), respectively, which could imply a different evolution pressure of the whole genome versus the core genome or a bias in codon usage.

### Whole-genome-based criteria

**FIGURE 4:**
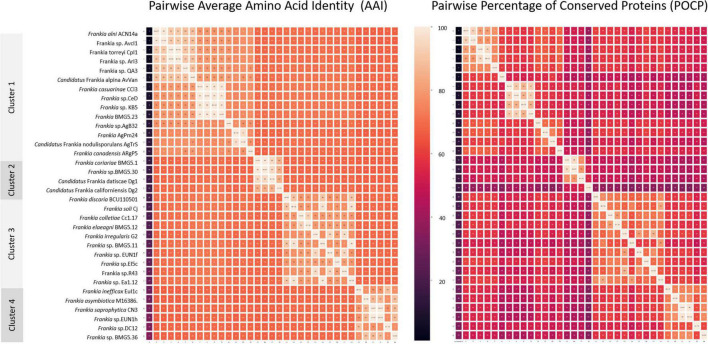
Heat maps based on pairwise average amino acid identity (AAI) and percentage of conserved proteins (POCP) calculated values. Pairwise AAI was calculated with the EzAAI tool (v1.1) using default settings, with a minimum query coverage of 50% and a minimum identity of 40%. The pairwise POCP was calculated considering an E-value of < 1 × 10–5, > 40% sequence identity and > 50% of the query sequence.

Inter-cluster pairwise AAI (Figure 4 and Supplementary Table 1), POCP (Figure 4 and Supplementary Table 2), and ANI (Supplementary Table 3) values ranged between 66.5 and 72.2, 33.5 and 61.3, and 78 and 81.5%, respectively. A cut-off AAI value at 72.2 ± 0.03%, which is within the 60–80% threshold recommended for the delineation of genera (Konstantinidis and Tiedje, 2005; Rodriguez-R and Konstantinidis, 2014), permitted a congruent regrouping with phylogenetic clusters. The POCP threshold of 50% originally proposed for genus delineation by Qin et al. (2014) has been shown to be inappropriate for multiple taxa (Aliyu et al., 2016; Pannekoek et al., 2016; Li et al., 2017; Lopes-Santos et al., 2017; Orata et al., 2018; Wirth and Whitman, 2018; Park et al., 2022). Sangal et al. (2022) considered this POCP cutoff as overly stringent and proposed its reappraisement to 58–66%. The inter-cluster pairwise POCP values obtained in this study (33.5–61.3%) are within this proposed new range.

When ANI values are compared between more divergent taxa than species, they are prone to saturation and loss of information and, hence, considered inappropriate standards for genus delineation (Konstantinidis and Tiedje, 2007; Kim et al., 2014; Qin et al., 2014; Rodriguez-R and Konstantinidis, 2014; Gosselin et al., 2022; Park et al., 2022). The ANI values in the present study (78–81.5%) were consistently lower than the ANI species thresholds of 95–96.5%. Values closer to the species threshold (89–90%) were, however, seen with the quartic function of 750 genomes analysed (Barco et al., 2020).

### Other genomic criteria

Some genes and operon organisation show some distinctiveness for each of the four clusters (Supplementary Figure 1). There are two ribosomal operons for clusters 1 and 2 strains and three for clusters 3 and 4 (Gtari et al., 2007). Nitrogenase complexes encoding (*nif*) genes are totally absent in the asymbiotic cluster 4 (not retrieved in the draft genome sequence, except for *F. asymbiotica* strain M16386^T^). The *nif* genes are, however, organised in different ways with respect to each cluster (Tisa et al., 2016; Nouioui et al., 2019b). The Ni-Fe hydrogenase or uptake hydrogenase (*hup*) genes are clustered into two operons for clusters 1 and 3, while only one operon is present for clusters 2 and 4 (Tisa et al., 2016). Another important distinctive gene is *murC*, related to peptidoglycan biosynthesis. Different copies in the genomes of the four different clusters were found (Berckx et al., 2020). Two copies, *mur*C1 and *mur*C2, are present in clusters 2 and 3, which differ in their orientation with the presence of an open reading frame (ORF) encoding a nitroreductase family deazaflavin-dependent oxidoreductase in cluster 3. Clusters 1 and 4 contain only one copy of *mur*C2 with the ORF encoding a nitroreductase family deazaflavin-dependent oxidoreductase only present in cluster 1.

**FIGURE 5:**
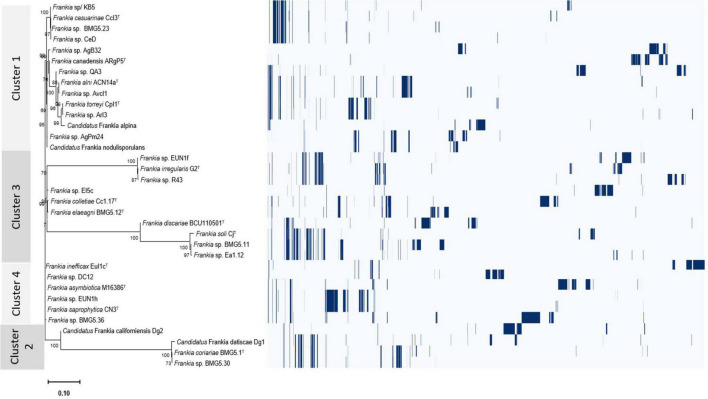
Roary plot showing the tree compared to a matrix with the presence and absence of core and accessory genes identified for the four cluster genomes.

Overall differentially encoded proteins between genomes of the four clusters were provided in Supplementary Table 4. The presence/absence profiles of the protein clusters for the four phylogenetic clusters were illustrated in Figure 5 and Supplementary Figure 2.

### Ecological and phenotypic features

The phylogenetic segregation of *Frankia* clusters found additional support in the ecological lifestyle interaction with host plants and cultural behaviours among the *Frankia* strains of each cluster. *Frankia* strains from cluster 1 (except those nodulating *Allocasuarina* and *Casuarina*) and cluster 3 are globally distributed in soils irrespective of the presence or absence of compatible host plants (Smolander and Sundman, 1987; Põlme et al., 2014). The distribution of cluster 1 of *Casuarinaceae* infective and cluster 2 strains is restricted to the native range of their respective host plants (Simonet et al., 1999). Cluster 4 ineffective strains (nodulating but non-nitrogen fixing) were shown to represent the most prominent *Frankia* population, exhibiting a higher diversity in prairie soils in the absence of actinorhizal host plants (Ben Tekaya et al., 2017) and wet soils under *Alnus glutinosa* (Hahn et al., 1988; van Dijk and Sluimer, 1994; Wolters et al., 1997a,b).

Most *Frankia* strains from clusters 1 and 3 have been cultured in axenic conditions with relative ease. Strains from cluster 4, which have been isolated as a “by-product” in studies aimed at the cultivation of the “true” beneficial microsymbionts in actinorhizal root nodules, show very similar cultural behaviours to other soil actinomycetes (Normand and Chapelon, 1997; Nouioui et al., 2017a,b, 2018a). Cluster 1 contains as-yet-uncultured *Frankia* microsymbionts, which are characterised by profuse sporulation within nodules tissues (Sp + types; Schwintzer, 1990), and a very higher proportion of cluster 2 strains are as-yet-uncultured. The *Candidatus* status has been used to accommodate some of these uncultured *Frankia* which were defined based on genome sequences (Persson et al., 2011, 2015; Nguyen et al., 2016, 2019; Normand et al., 2017; Herrera-Belaroussi et al., 2020; Pozzi et al., 2020; Berckx et al., 2022). Two strains from cluster 2 have been successfully cultivated following a dual approach consisting of comparative genomics and direct physiological assay on nodule tissues (Gtari et al., 2015; Gueddou et al., 2019).

While filamentous hyphae are the primary vegetative state for all growing *Frankia* strains, the extent of sporangia and vesicle formation varies from cluster to cluster. The sporulation of strains from clusters 1, 3, and 4 may be readily detected *in vitro* or may depend on the composition of media and cultural conditions (Tisa et al., 1983; Krumholz et al., 2003). The sporulation of the two cultivated strains of cluster 2 seems to be completely suppressed (Gtari et al., 2015; Nouioui et al., 2017b). In general, vesicles containing nitrogenase are formed in response to the limited availability of nitrogen (Fontaine et al., 1984; Murry et al., 1984). Some *Frankia* strains, belonging to cluster 3, continue to form vesicles even in the presence of a nitrogen source, but the numbers are reduced compared with growth in nitrogen-depleted media (Gauthier, 1983; Meesters et al., 1985). Strains of cluster 4 are unable to fix dinitrogen and thus to form vesicles, with the exception of *F. asymbiotica*.

Strains from clusters 1 and 3 grow well in nitrogen-depleted media and metabolise short-chain fatty acids, TCA cycle intermediates, and carbohydrates. Strains in cluster 4 are similar to other saprophytic actinomycetes, more active physiologically, grow more rapidly and utilise a variety of monosaccharides and disaccharides, and produce hydrolytic enzymes, such as pectinases, cellulases, amylases, and proteases (Lechevalier, 1994). The two cultivated strains from cluster 2 are more slowly growing and have an unusual physiological requirement for alkalophilic growth media (Gtari et al., 2015; Nouioui et al., 2017b). Other phenotypic markers, including chemotaxonomy, are provided in Table 2.

**TABLE 2:** Phenotypic and host-plant-related features.

	Cluster 1 species				Cluster 2 species	Cluster 3 species					Cluster 4 species		
	ACN14A^T^	CpI1^T^	ARgP5^T^	CcI3^T^	BMG5.1^T^	BMG5.12^T^	BCU110501^T^	G2^T^	Ci^T^	Cc1.17^T^	EuI1c^T^	M16386^T^	CN3^T^
Colony colour	White	White	White	White	Brown	Red	Yellow	Red	Yellow	Yellow	White	White	White greyish
Vesicles/N_2_-fixation	+	+	+	+	+	+	+	+	+	+	−	+	−
Sporangia	+	+	+	+	−	+	+	+	+	+	+	+	+
Major fatty acids (>15%)	*iso*-C_16:0_, C_17:1_ ω8c	*iso*-C_16:0_, C_17:1_ ω8c	*iso*-C_16:0_, C_17:1_ω8c	*iso*-C_16:0_, C_17:1_ ω8c	C_18:1_ω9c, C_16:0_	*iso*-C_16:0_, C_16:0_, C_17:1_ω8c	*iso*-C_16:0_, C_17:1_ω8c, C_16:0_	*iso*-C_16:0_, C_17:1_ω8c, C_15:0_	*iso*-C_16:0_, C_17:1_ ω8c	C_16:0_, iso-C_16:0_, C_17:1_ ω9, C_18:1_ω9	*iso*-C_16:0_, C_17:1_ω8c, C_17:0_,	*iso*-C_16:0_, C_17:1_ ω8c	*iso*-C_16:0_, C_17:1_ ω8c, C_15:0_
Predominant menaquinones (>20%)	MK-9(H_8_), MK-9(H_4_)	MK-9(H_8_)	MK-9(H_8_)	MK-9(H_6_), MK-9(H_8_)	MK-9(H_6_), MK-9(H_4_)	MK-9(H_4_), MK-9(H_6_)	MK-9(H_4_)	MK-9(H_4_); MK-9(H_6_)	MK-9(H_4_)		MK-9(H_6_), MK-9(H_4_)	MK-9(H_4_), MK-9(H_6_)	MK-9(H_6_)
Phospholipid^1^	PI, DPG, GPL1–3, PG (L).	PI, DPG, PG, APL (PL) (L)	DPG, PI PG	PI, DPG, GPL1–3, PG (L).	PI, DPG, PG GLs	PI, DPG, GPL1–3, PG (L)	PI, DPG, GPL1-3, PG (L)	PI, DPG, GPL1–2, PG, APL (L)	PI, PG, DPG GL	PI, DPG, PG GPL GPL1-2, GL1-5	PI, DPG, PG (GPL1-2)	PI, PG, DPG, GPL, PL	PI, PG, DPG, PL (GL1-6), (L)
Cell wall sugars	Galactose, glucose, mannose, rhamnose, ribose, and xylose	Galactose, glucose, mannose, rhamnose, ribose, and xylose	Galactose, glucose, mannose, rhamnose (trace), ribose, and xylose	Galactose, glucose, mannose, rhamnose, ribose, and xylose	Galactose, glucose, mannose, and a trace of ribose	Galactose, glucose, mannose, rhamnose, ribose, and xylose	Galactose, glucose, mannose, xylose, and ribose	Galactose, glucose, mannose, rhamnose, ribose, and xylose	Galactose, glucose, mannose, xylose, ribose, and a trace of rhamnose	Galactose, glucose, mannose, rhamnose, ribose, and xylose	Glucose, galactose, mannose, ribose, rhamnose, and fucose	Galactose, glucose, mannose, rhamnose, and ribose	Galactose, glucose, mannose, rhamnose, and ribose (in traces)
Host specifity group^2^	HSG1			HGS2	HSG5	HSG3/HGS4					HSG3	–	
Mode of infection^3^	RHI				CE/IC	IC/CE						–	

**^1^**DPG, diphosphatidylglycerol; GL, glycolipid; GPL, unknown glycophospholipid; PG, phosphatidylglycerol; PI, phosphatidylinositol; PL, phospholipids; UL, unidentified lipids. Phospholipid between brackets is yet uncharacterized. ^2^HSG1 infecting *Alnus (Betulaceae), Comptonia, Morella*, and *Myrica (Myricaceae)* species; HSG2 infecting *Casuarina* and *Allocasuarina (Casuarinaceae)* and *Morella* species; HSG3 infecting *Elaeagnaceae, Colletieae (Rhamnaceae), Gymnostoma (Casuarinaceae)*, and *Morella (Myricaceae)* species; HSG4 strains nodulate members of the *Elaeagnaceae* but not the promiscuous hosts in the *Myricaceae* or *Gymnostoma (Casuarinaceae)*; HSG5, strains nodulate members of *Coriariaceae, Datiscaceae, Dryadoideae (Rosaceae)*, and *Ceanothus (Rhamnaceae)* species. ^3^RH, root-hair infection; CE; crack entry, IC; intercellular (Nguyen and Pawlowski, 2017).

### Conclusion and description of the new taxa

Evidence of the splitting of *Frankia* into novel genera is here provided based on phylogenomics and OGRI recommended for bacterial genus boundary demarcation. The taxonomic elevation of phylogenetically distant *Frankia* clusters is clearly supported through the consistent sequence divergence in phylogenetic trees, OGRI analysis, and other genome-related criteria. The genus *Frankia* should be revised to accommodate cluster 1 species only, while clusters 2, 3, and 4 are taxonomically elevated to the rank of the genus as *Protofrankia* gen. nov., *Parafrankia* gen. nov., and *Pseudofrankia* gen. nov., respectively, and therefore, new combinations are provided for related species names. In the case of *Protofrankia*, in addition to the type species, two previously described *Candidatus* species can be reclassified as members of the genus. Thus, *Candidatus* Frankia datiscae (Persson et al., 2011) should be renamed *Candidatus* Protofrankia datiscae and *Candidatus* Frankia californiensis (Normand et al., 2017) should be renamed *Candidatus* Protofrankia californiensis.

The taxonomic revision provided, in this study, will help clarify the confusing past classification of the actinorhizal microsymbionts for taxonomic and applied purposes.

### Description of *Protofrankia* gen. nov.

*Protofrankia* (Pro.to.fran’ki.a. Gr. masc. adj. *protos*, earlier than, prior to; N.L. fem. n. *Frankia* a bacterial genus name; N.L. fem. n. *Protofrankia*, a genus considered here as phylogenetically basal to *Frankia*).

The genus is defined by the taxonomic elevation of the taxon previously defined as *Frankia* phylogenetic cluster 2. Host plants include *Coriariaceae*, *Datiscaceae*, *Dryadoideae* (*Rosaceae*), and *Ceanothus* (*Rhamnaceae*) species. Genome sizes in the range of 5.3–5.8 Mb with G + C mol% content of 68.0–70.2%. The type species is *Protofrankia coriaria*.

### *Protofrankia coriaria* comb. nov.

*Protofrankia coriaria* (co.ri.a’ri.ae. N.L. gen. fem. n. *coriariae*, of *Coriaria*, referring to the origin of isolation of the type strain).

Basonym: *Frankia coriaria* (Nouioui et al., 2017b,c).

The description of *Protofrankia coriaria* comb. nov. is identical to that given by Nouioui et al. (2017b) for *F. coriaria*. The type strain is BMG5.1^T^ (= DSM 100624^T^ = CECT 9032^T^).

### Description of *Parafrankia* gen. nov.

*Parafrankia* (Pa.ra.fran’ki.a. Gr. prep. *para*, beside; N.L. fem. n. *Frankia*, a bacterial genus name; N.L. fem. n. *Parafrankia*, beside *Frankia*).

The genus *Parafrankia* is defined by the taxonomic elevation of the taxon previously defined as *Frankia* phylogenetic cluster 3. Host plants include members of *Elaeagnaceae*, *Colletieae*, *Morella*, and *Gynmnostoma*. Genome sizes range from 6.6 to 11.2 Mb with G + C mol% of 69.7–72.3. The type species is *Parafrankia elaeagni*. In addition, four species can be reclassified as members of the genus. *Frankia discariae* (Nouioui et al., 2018b), *F. irregularis* (Nouioui et al., 2018a), *F. soli* (Gtari et al., 2020), and *F. colletiae* (Nouioui et al., 2022) should be named *Parafrankia discariae*, *Parafrankia irregularis*, *Parafrankia soli*, and *Parafrankia colletiae*, respectively.

### *Parafrankia elaeagni* comb. nov.

*Parafrankia elaeagni* (e.lae.ag’ni. N.L. gen. masc. n. *elaeagni*, of *Elaeagnus*, referring to the source of the isolate).

Basonym: *Frankia elaeagni* (Nouioui et al., 2016).

The description of *Parafrankia elaeagni* is the same as that given by Nouioui et al. (2016) for *F. elaeagni*. The type strain is BMG5.12^T^ (= DSM 46783^T^ = CECT 9031^T^).

### *Parafrankia discariae* comb. nov.

*Parafrankia discariae* (dis.ca’ri.ae. N.L. gen. fem. n. *discariae*, of *Discaria*, the host plant origin of isolation of the type strain).

Basonym: *Frankia discariae* (Nouioui et al., 2017d).

The description of *Parafrankia discariae* is the same as that given by Nouioui et al. (2017d) for *F. discariae*. The type strain is BCU110501^T^ (= DSM 46785^T^ = CECT 9042^T^).

### *Parafrankia irregularis* comb. nov.

*Parafrankia irregularis* (ir.re.gu.la’ris. L. fem. adj. *irregularis*, of irregular, referring to the inability of the species to infect its original host plant and to infect taxonomically disparate host plants).

Basonym: *Frankia irregularis* (Nouioui et al., 2018b).

The description of *Parafrankia irregularis* is the same as that given by Nouioui et al. (2018b) for *F. irregularis*. The type strain is G2^T^ (= DSM 45899^T^ = CECT 9038^T^).

### *Parafrankia soli* comb. nov.

*Parafrankia soli* (so’li. L. gen. neut. n. *soli*, of soil, referring to the isolation source of the type strain).

Basonym: *Frankia soli* (Gtari et al., 2020).

The description of *Parafrankia soli* comb. nov. is the same as that given by Gtari et al. (2020) for *F. soli.* The type strain is Cj^T^ (= DSM 100623^T^ = CECT 9041^T^ = NRRL B-16219^T^).

### *Parafrankia colletiae* comb. nov.

*Parafrankia colletiae* (col.le’ti.ae. N.L. gen. n. colletiae of Colletia, referring to the host plant, *Colletia*, origin of isolation of the strain).

Basonym: *Frankia colletiae* (Nouioui et al., 2022).

The description of *Parafrankia colletiae* comb. nov. is the same as that given by Nouioui et al. (2022) for *F. colletiae*. The type strain is Cc1.17^T^ (= DSM 43829^T^ = CECT 9313^T^).

### Description of *Pseudofrankia* gen. nov.

*Pseudofrankia* (Pseu.do.fran’ki.a. Gr. masc. adj. *pseudes*, false; N.L. fem. n. *Frankia*, a bacterial genus name; N.L. fem. n. *Pseudofrankia*, a false *Frankia*).

*Pseudofrankia* gen. nov. is defined by the taxonomic elevation of the taxon previously defined as *Frankia* phylogenetic cluster 4. Members of the genus have been isolated from actinorhizal root nodules and are non-infective and/or non-nitrogen-fixing taxa. The size range of the genomes is 6.6–9.9 Mb with 71.2–72.3 of G + C mol%. The type species is *Pseudofrankia inefficax*. In addition, two other species can be reclassified as members of the genus. *Frankia asymbiotica* (Nouioui et al., 2018a) and *F. saprophytica* (Nouioui et al., 2018) should be named *Pseudofrankia asymbiotica* and *Pseudofrankia saprophytica*, respectively.

### *Pseudofrankia inefficax* comb. nov.

*Pseudofrankia inefficax* (in.ef’fi.cax. L. fem. adj. *inefficax*, inefficient in reference to the inability of the bacterium to form the effective nitrogen-fixing symbiosis with its plant host).

Basonym: *Frankia inefficax* (Nouioui et al., 2017a).

The description of *Pseudofrankia inefficax* comb. nov. is the same as that given by Nouioui et al. (2017a) for *F. inefficax*. The type strain is EuI1c^T^ (=DSM 45817^T^ = CECT 9037^T^).

### *Pseudofrankia asymbiotica* comb. nov.

*Pseudofrankia asymbiotica* (a.sym.bi.o’ti.ca. Gr. pref. *a-*, not; N.L. fem. adj. *symbiotica*, living together; N.L. fem. adj. *asymbiotica*, not symbiotic).

Basonym: *Frankia asymbiotica* (Nouioui et al., 2017c).

The description of *Pseudofrankia asymbiotica* comb. nov. is the same as that given by Nouioui et al. (2017c) for *F. asymbiotica*. The type strain is M16386^T^ (= DSM 100626^T^ = CECT 9040^T^ = NRRL B-16386^T^).

### *Pseudofrankia saprophytica* comb. nov.

*Pseudofrankia saprophytica* (sa.pro.phy’ti.ca. Gr. masc. adj. *sapros*, rotten; Gr. masc. adj. *phytikos*, belonging to plants; N.L. fem. adj. *saprophytica*, growing on rotten material, referring to the asymbiotic lifestyle of the type strain).

Basonym: *Frankia saprophytica* (Nouioui et al., 2018a).

The description of *Pseudofrankia saprophytica* comb. nov. is the same as that given by Nouioui et al. (2018a) for *F. saprophytica*. The type strain is CN3^T^ (=DSM 105290^T^ = CECT 9314^T^).

## Data availability statement

The original contributions presented in this study are included in the article/Supplementary material, further inquiries can be directed to the corresponding author.

## Author contributions

MG conceived the study, performed the analyses, and wrote the manuscript.

## Footnotes

1
https://tygs.dsmz.de/

## Supplementary material

Supplementary Figure 1 Comparative representations of the *nif* operons in strains representing *Frankia* clusters 1, 2, and 3 **(A)**, and *hup* gene cluster **(B)** and biosynthetic peptidoglycan genes *mur*C **(C)** in clusters 1, 2, 3, and 4.

Supplementary Figure 2 Clusters of Orthologous Genes (COG) categories distribution for all studied strains.

## Abbreviations

AAI: average amino acid identity
ANI: average nucleotide identity
GGDC: genome-to-genome distance calculator
ML: maximum-likelihood
MP: maximum-parsimony
AFLP: amplified fragment length polymorphism
MLSA: multilocus sequence analysis
POCP: percentage of conserved proteins
TYGS: type strain genome server
LPSN: list of prokaryotic names with standing in nomenclature.
