# Supplementary figures and images for: Taxogenomic status of phylogenetically distant Frankia clusters warrants their elevation to the rank of genus: A description of Protofrankia gen. nov., Parafrankia gen. nov., and Pseudofrankia gen. nov. as three novel genera within the family Frankiaceae
Source: Front Microbiol. 2022 Nov 8;13:1041425. doi: 10.3389/fmicb.2022.1041425 (PMC9680954; doi:10.3389/fmicb.2022.1041425)

a

Cluster 1

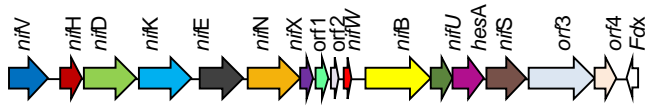

Cluster 2

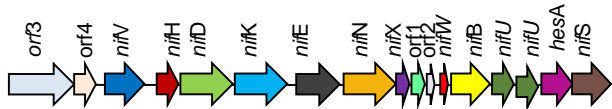

Cluster 3

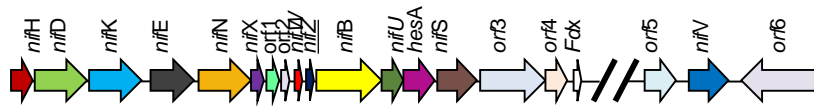

c

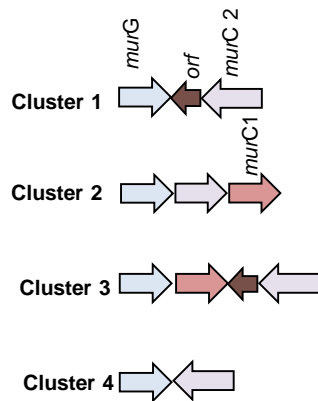

b

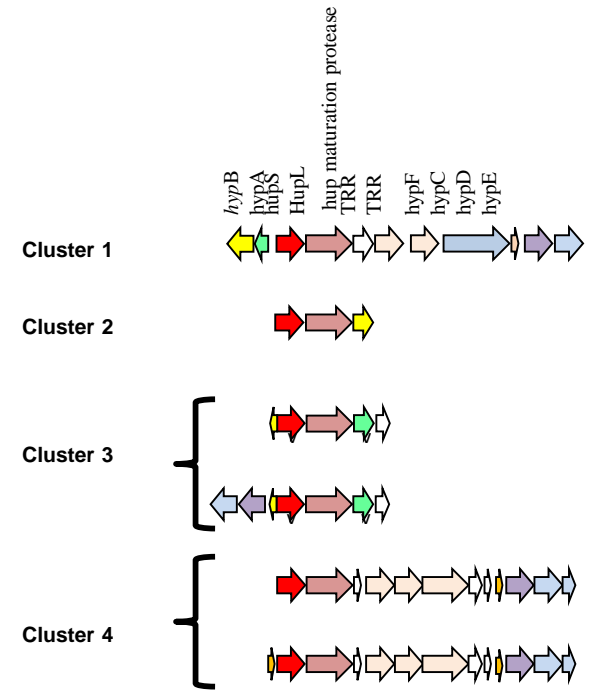

Supplement: Supplementary Figure 1 — Comparative representations of the nif operons in strains representing Frankia clusters 1, 2, and 3 (A), and hup gene cluster (B) and biosynthetic peptidoglycan genes murC (C) in clusters 1, 2, 3, and 4. [file Data_Sheet_1.PDF]

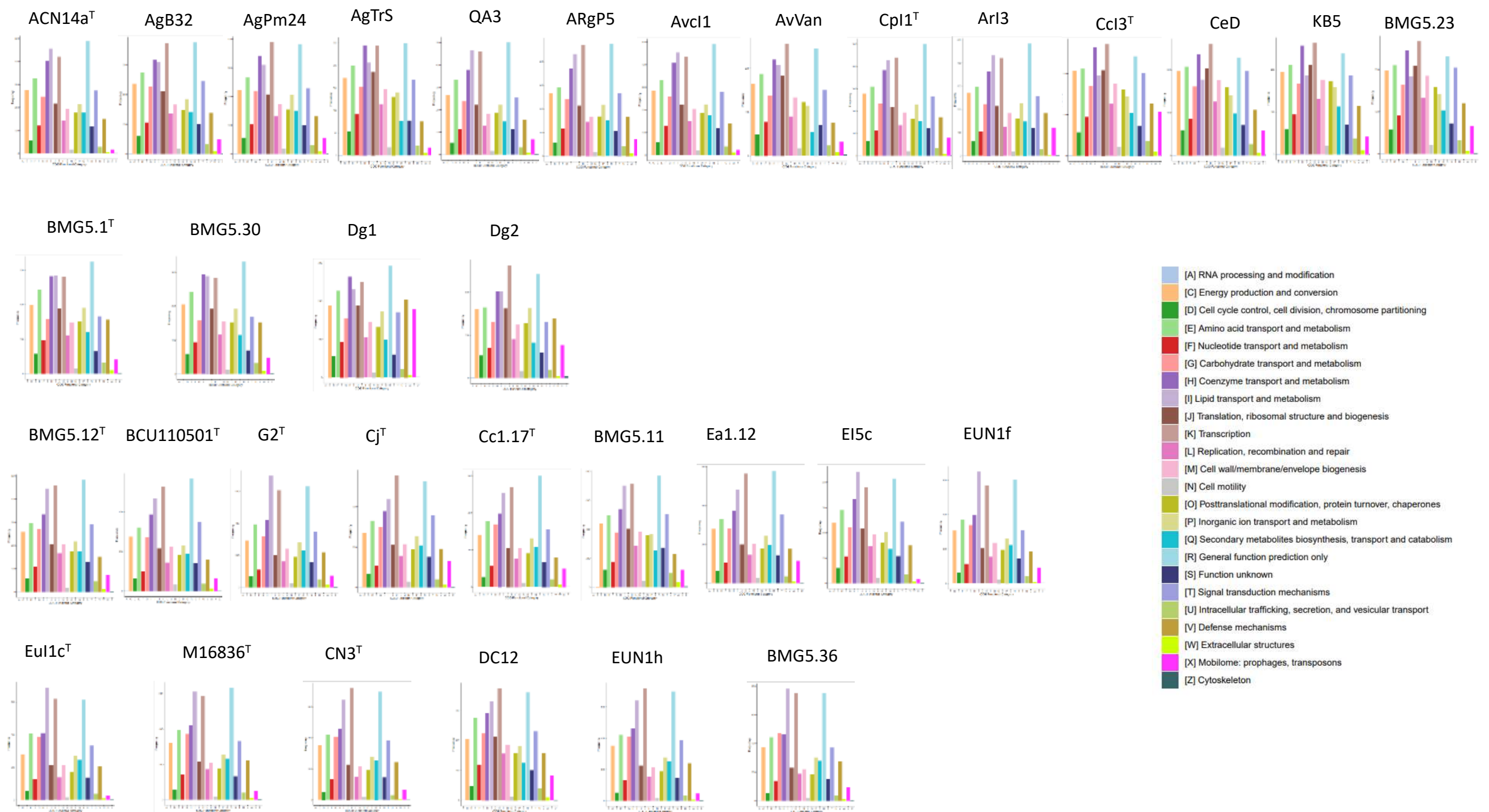

Supplement: Supplementary Figure 2 — Clusters of Orthologous Genes (COG) categories distribution for all studied strains. [file Data_Sheet_2.PDF]
